# Identification of High-Order Single-Nucleotide Polymorphism Barcodes in Breast Cancer Using a Hybrid Taguchi-Genetic Algorithm: Case-Control Study

**DOI:** 10.2196/16886

**Published:** 2020-06-17

**Authors:** Li-Yeh Chuang, Cheng-San Yang, Huai-Shuo Yang, Cheng-Hong Yang

**Affiliations:** 1 I-Shou Uneiversity Kaohsiung City Taiwan; 2 Ditmanson Medical Foundation Chia-Yi Christian Hospital Chiayi City Taiwan; 3 Department of Electronic Engineering National Kaohsiung University of Science and Technology Kaohsiung City Taiwan; 4 Drug Development and Value Creation Research Center Kaohsiung Medical University Kaohsiung Taiwan; 5 College of Medicine Kaohsiung Medical University Kaohsiung Taiwan

**Keywords:** genetic algorithm, single-nucleotide polymorphism, breast cancer, case-control study

## Abstract

**Background:**

Breast cancer has a major disease burden in the female population, and it is a highly genome-associated human disease. However, in genetic studies of complex diseases, modern geneticists face challenges in detecting interactions among loci.

**Objective:**

This study aimed to investigate whether variations of single-nucleotide polymorphisms (SNPs) are associated with histopathological tumor characteristics in breast cancer patients.

**Methods:**

A hybrid Taguchi-genetic algorithm (HTGA) was proposed to identify the high-order SNP barcodes in a breast cancer case-control study. A Taguchi method was used to enhance a genetic algorithm (GA) for identifying high-order SNP barcodes. The Taguchi method was integrated into the GA after the crossover operations in order to optimize the generated offspring systematically for enhancing the GA search ability.

**Results:**

The proposed HTGA effectively converged to a promising region within the problem space and provided excellent SNP barcode identification. Regression analysis was used to validate the association between breast cancer and the identified high-order SNP barcodes. The maximum OR was less than 1 (range 0.870-0.755) for two- to seven-order SNP barcodes.

**Conclusions:**

We systematically evaluated the interaction effects of 26 SNPs within growth factor–related genes for breast carcinogenesis pathways. The HTGA could successfully identify relevant high-order SNP barcodes by evaluating the differences between cases and controls. The validation results showed that the HTGA can provide better fitness values as compared with other methods for the identification of high-order SNP barcodes using breast cancer case-control data sets.

## Introduction

Breast cancer has a major disease burden in the female population, with a growing incidence recently [1,2]. Previously, several interpretations of associations between breast cancer and tumor characteristics [3-5], single-nucleotide polymorphisms (SNPs) [6-8], clinicopathological factors [9], and biomarkers [10] revealed relevant association effects between these factors and the risk of cancer. Previous studies also indicated that genomic variation could contribute to the tumorigenicity process in breast cancer [11-14]. Thus, effective approaches for breast cancer estimation are required.

SNPs are crucial genetic variants in genomic association analyses involving leukemia [15], cancers [16], and other diseases [17-19]. Numerous SNPs cannot be excluded from analyses as no relevant differences between cases and controls can be found through conventional methods. Some SNPs may have relevant associations with other SNPs, and these associations are referred to as SNP barcodes. Consequently, the detection of SNP barcodes is vital for association analyses of diseases and cancers [20-23].

An SNP barcode consists of SNPs, and each SNP includes three genotypes. The large space of suitable SNP barcode combinations complicates the statistical evaluation and identification of relevant SNP barcodes. Evolutionary algorithms have been proposed to facilitate statistical identification of SNP barcodes, and a genetic algorithm (GA) is one of the most frequently used algorithms in genomic studies [24,25]. A GA is an effective approach in the identification of relevant genetic associations for various diseases through the use of more efficient search abilities to enhance population diversity [26]. The crossover and local search operations in a GA can reduce the probability of the same vector being identified between two selected SNPs, and hence, they can improve the search ability of this algorithm.

Breast cancer is a major health issue, and machine learning algorithms are frequently employed to detect the complex genomic associations in breast cancer studies. Although previous machine learning approaches could effectively identify SNP associations in genomic studies, the detection rate of SNP barcodes remains challenging for high-order SNP barcodes. Thus, we proposed a hybrid Taguchi-genetic algorithm (HTGA) for high-order SNP barcode identification in a breast cancer case-control study.

## Methods

### Genetic Algorithm

A GA is a machine learning algorithm inspired by biological evolutionary processes [27]. The first GA operation is population initialization, in which solutions are produced over the solution space; these initial solutions are designated as parents. In the population, two parents are strategically selected according to some fitness values for crossover operators. Crossover operators generate offspring by combining the chromosomal matter from the two parents. Mutation operations can increase population diversity through localized change, eliminating inferior chromosomes from the population and retaining good offspring. Thus, the good factors within the population can be passed on to the next generation. The aforementioned operations and population replacement are repeated until the stopping criterion is satisfied.

### Taguchi Method

The methods proposed by Taguchi et al [28] are based on a statistical experimental design to improve the evaluation and performance of products, process conditions, and parameter settings. Taguchi methods primarily rely on orthogonal arrays (OAs) and the signal-to-noise ratio (SNR). An OA is a fractional factorial matrix that provides a comprehensive analysis of interactions among all design factors. This matrix ensures a proportionate comparison of levels for all factors. A two-level OA can be defined as *L_n_* (2*^n^*^−1^), where *n*=2*^k^* is the number of experimental runs, *k* (1) is a positive integer, base 2 represents two levels for each design parameter, and *n*−1 is the number of columns in the OA. “*L”* represents “Latin,” because the OA experimental design concept is associated with the Latin square. An example of an OA is shown in Table 1.

SNR (η) is used as the selection quality characteristic in the field of communications engineering; it can be used to optimize the parameters for a target. Taguchi methods can classify the parameter design problem into several categories according the problem. Both smaller-the-better and larger-the-better SNR types are used. Considering the set of characteristics y_1_, y_2_, …, y_n_, in the smaller-the-better case, the SNR can be determined using the following equation:

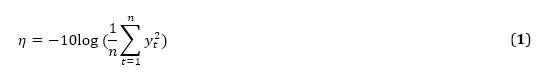



In the larger-the-better case, the SNR can be determined using the following equation:

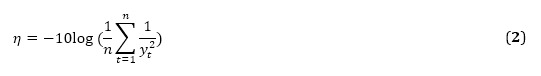



The SNR evaluates the robustness of the levels of each design parameter. A high-quality result can be achieved for a particular target by controlling the parameters at a particular level with a high SNR value.

**Table 1:** A L8(27) orthogonal array.

Experiment number	Factors						
	A	B	C	D	E	F	G
1	1	1	1	1	1	1	1
2	1	1	1	2	2	2	2
3	1	2	2	1	1	2	2
4	1	2	2	2	2	1	1
5	2	1	2	1	2	1	2
6	2	1	2	2	1	2	1
7	2	2	1	1	2	2	1
8	2	2	1	2	1	1	2

### Hybrid Taguchi-Genetic Algorithm

**Figure 1:**
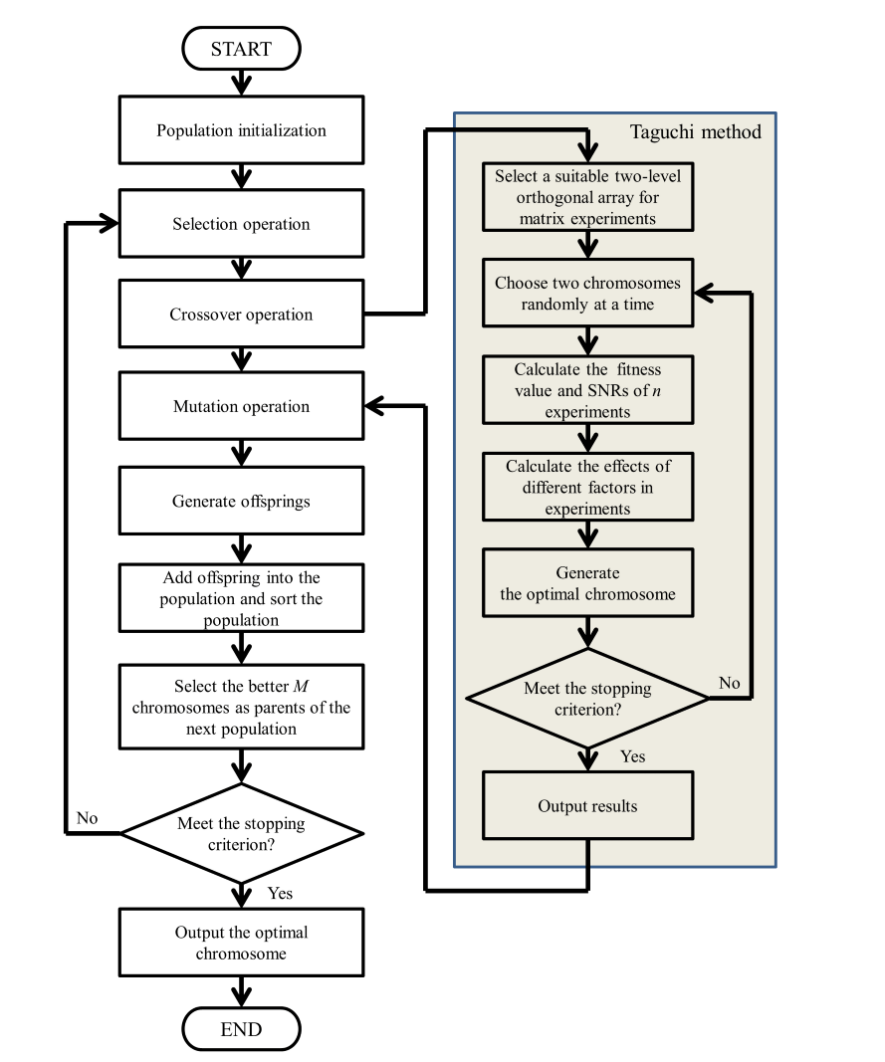
Hybrid Taguchi-genetic algorithm flowchart. SNR: signal-to-noise ratio.

In the HTGA, a Taguchi method is added into GA crossover and mutation operations. Figure 1 depicts a flowchart of the HTGA approach, which includes the below-mentioned 17 steps. The pseudocode of the HTGA is shown in Textbox 1.

#### HTGA Procedure

The procedure involves the following 17 steps: (1) Population initialization, execute the algorithm and generate an initial population; (2) Fitness value evaluations, evaluate the population’s fitness values; (3) Selection operation, select candidates using the tournament approach; (4) Crossover operation, the probability of crossover is determined by the crossover rate *p_c_*; (5) Select a suitable two-level orthogonal array for the experiment; (6) Randomly choose two chromosomes at a time to execute a matrix experiment; (7) Calculate the function values and SNRs of *n* experiments in the orthogonal array *L_n_* (2*^n^*^−1^); (8) Calculate the effects of different factors and in the experiment; (9) An optimal chromosome is generated; (10) Repeat steps 5 through 8 until the expected number (1/2) × *M* × *p_c_* is reached; (11) New chromosomes are generated through the Taguchi method; (12) Mutation operation, mutation probability is determined by the mutation rate *p*_m_; (13) Add chromosomes from a pool into the population; (14) Sort the population by fitness; (15) Select the fittest chromosomes as the new population for the next generation; (16) If the stopping criterion is met, execute step 17; if not, go back to step 2; (17) The chromosome with the highest fitness value is the HTGA solution.

**Pseudocode of the hybrid Taguchi-genetic algorithm.**

**Input:** maximum iteration as *T* (termination criterion)

     population size *M*

     crossover rate *P_c_*

     mutation rate *P_m_*

**Output:** optimal chromosome (the optimal solution)

**begin**

# *Initialization*

/* Initialize *M* chromosomes as *population* */

**for***iteration* ← 1 to *T* **do**

     # *Selection operation*

     **for***i* ← 1 to *M* **do**

         /* Randomly select two chromosomes from *population* as *chromosome*_1_ and

         *chromosome*_2_ */

         **if** *fitness*_1_ ≥ *fitness*_2_ **do**

             *winner* ← *chromosome*_1_

         **end if**

         **else do**

             *winner* ← *chromosome*_2_

         **end if**

             /* put *winner* into *mating pool* */

     **end for**

     # *Crossover operation*

     **for***i* ← 1 to (*M* / 2) **do**

         /* Sequentially select two chromosomes from *mating pool* as *chromosome*_1_

         and *chromosome*_2_ */

         **if** random() < *P_c_* **do**

             crossover(*chromosome*_1_, *chromosome*_2_)

             /* generate two offspring */

         **end if**

         /* put two offspring into *offspring pool* */

     **end for**

     *# Taguchi operation*

     **for***i* ← 1 to (0.5 × *M* × *P_c_*) **do**

         /* Randomly select two chromosomes from *offspring pool* as *chromosome*_1_

         and *chromosome*_2_ */

         Taguchi(*chromosome*_1_, *chromosome*_2_)

         /* generate one offspring */

         /* put one offspring into *offspring pool* */

     
**end for**

     # Mutation operation

     **for***i* ← 1 to size of *offspring pool* **do**

         **for***j* ← 1 to dimensions of chromosome **do**

             **if** random() < *P_m_* **do**

                 mutation(chromosome[*j*])

             **end if**

         **end for**

         /* generate one offspring */

         /* put one offspring into *offspring pool* */

     
**end for**

     # Replacement operation

     /* Reserve best *M* chromosomes as new *population* from *population* and *offspring*

     *pool* */

**end for**

/* Obtain optimal chromosome */

**End**

#### Encoding Schemes and Population Initialization

In the proposed GA, a suitable solution to a problem is denoted as chromosome *C* = {*c*_1_, *c*_2_, …, *c_n_*}, and the encoding scheme aims to design suitable elements in a chromosome. In the SNP barcode problem, the elements in a chromosome include (1) the indexes of the selected SNPs in the data set and (2) the genotypes of these selected SNPs. Thus, a chromosome *C_i_* is expressed as shown in equation 3.

*C_i_* = (*SNP_i,s_*, *Genotype_i,g_*) (**3**)

where *i* = 1, 2, …, *m*, and is the population size. *SNP_i,s_*, where *s* = 1, 2, …, *n*/2, is a selected SNP dimension in which all SNPs are unrepeatable, and *n* is the SNP barcode order. *Genotype_i,g_* represents the three possible genotypes of the selected *SNP_i,s_*, where *g* = *n*/2 + 1, *n*/2 + 2, …, *n* is the selected genotype dimension. In the population initialization, all chromosomes are stochastically generated according to the encoding schemes.

#### Fitness Function Evaluation

The aim of SNP barcode identification is to detect relevant differences between cases and controls. To optimize the protective effect of the SNP combination, a fitness function is required for comparing cases and controls. A high difference between cases and controls indicates a high probability of detecting relevant SNP barcodes. In the proposed GA, a chromosome is measured by the fitness function shown in equation 4.

*F*(*C_i_*) = *number* (*control*∩*C_i_*) − *number* (*case*∩*C_i_*) (**4**)

where *number* is the total number of elements in a set, *control* denotes the controls, *case* denotes the cases, and *C_i_* is the *i*th chromosome. Thus, the number of intersections between the *i*th chromosome and the controls is calculated by *number* (*control*∩*C_i_*), and the number of intersections between the *i*th chromosome and the cases is calculated by *number* (*case*∩*C_i_*). Thereafter, we calculate the difference between *number* (*control*∩*C_i_*) and *number* (*case*∩*C_i_*) as the fitness value at *C_i_*.

#### Selection Operation

In the selection operation, a random tournament selection scheme is used to pick each pair of parents from the population [29]. In tournament selection, two chromosomes are randomly selected to compare their individual fitness values. The chromosomes with better fitness values are inserted into the mating pool. According to the mechanism of tournament selection, the probability that the average fitness value of solutions in the mating pool is better than the average fitness value of the parent population is high. Chromosomes in the mating pool are selected for the crossover operation and used to produce offspring. Textbox 2 provides the pseudocode of tournament selection. The selection operation is repeatedly executed until the maximum mating pool size is achieved.

**Tournament selection procedure.**

**Input:***population*, the list of chromosomes to select from

**Input:***chromosome*_1_, the first randomly selected chromosome from population

**Input:***chromosome*_2_, the second randomly selected chromosome from population

**Input:***fitness*_1_, the fitness value of the first chromosome

**Input:***fitness*_2_, the fitness value of the second chromosome

**Output:***winner*: the chromosome with better fitness value in tournament

**Output:***mating pool*: reserve the list of chromosomes to execute crossover operation

*# Tournament selection*

**begin**

**for***i* ← 1 to size of mating pool **do**

     Randomly select two chromosomes from *population*

     **if** *fitness*_1_ ≥ *fitness*_2_ **do**

         *winner* ← *chromosome*_1_

     **end if**

     **else do**

         *winner* ← *chromosome*_2_

     
**end else**

     put *winner* into *mating pool*

**end for**

#### Crossover Operation

After the selection operation, the crossover operation is implemented to create high-performing individuals. Two chromosomes are sequentially selected from the mating pool as a pair of parents, and then, the crossover operation is executed on them. The crossover operation uses a uniform crossover. Each bit in a chromosome is randomly generated as 0 or 1, and for 1, points are swapped between parent organisms; otherwise, points are not swapped. The encoding schemes establish a single point as an SNP locus with a corresponding genotype locus at the *j* 2 + 1 position, where *j* = 1, 2, …, *n*/2 is the index in the chromosome and *n* is the SNP barcode order. Therefore, *n*/2 bits are randomly generated, and both the *j* 2 + 1 genotype locus and *j*th bit representing an SNP are swapped in the parent organisms.

#### Taguchi Operation

An orthogonal array exhibits *Q* design factors. Each factor has two levels. An orthogonal array *L_n_* (2*^n^*^−1^) exhibits *n*−1 columns and *n* individual experiments corresponding to *n* rows, where *n* = 2^k^ and *Q* ≤ *n*−1; *k* is a positive integer, defined as an integer >1, and it is used for adjusting the number of experimental runs.

The SNR (*η*) is the mean square deviation of the fitness function. Let two values of *η* be *η_i_* = (*y_i_*)^2^ and *η_i_* = −(*y_i_*)^2^ (where is negative) in the case of a fitness function that is maximized (larger-the-better). Let *y_i_* be the function evaluation value of experiment *i* = 1, 2, 3, …, *n*, where *n* is the number of experiments. The effect of factor *f* is defined as follows:

*E_fl_* = sum of *η_i_* for factor *f* at level *l* (**5**)

where *i* is the experiment number, *f* is the factor name, and *l* is the level number.

#### Mutation Operation

The mutation operation aims to prevent the population from falling into local optima. In all suitable solutions, each offspring element has a chance to undergo a mutation operation. Each mutation position with a probability of mutation *p_m_* generates a random number in (0, 1). If the number is less than *p_m_* at the *i*th element in an offspring specimen, the *i*th element will be mutated by a randomly generated possible value.

#### Replacement Operation

The replacement operation uses an individual to replace the weakest individual in the population. After the completion of the aforementioned operations, the offspring are added to the population, and then, all the parents and offspring are ranked based on their fitness values. Subsequently, top *p* chromosomes in the population size are selected as the new population for the next generation, where *p* is the population size.

#### Termination Condition

The HTGA operation is repeated in successive iterations until the stopping criterion is met. In this study, a maximum number of iterations was used to terminate HTGA operations.

### Parameter Setting

This study compared the search effectiveness of the HTGA with that of standard GA, particle swarm optimization (PSO) [30], and chaotic PSO (CPSO) [31] methods. PSO is a swarm intelligence algorithm that simulates the social behavior of organisms. In PSO, each individual represents a particle and considers a potential solution in the swarm population. In CPSO, chaotic theory is incorporated into PSO to increase the search space and enhance PSO performance. PSO and CPSO parameters include population size, iteration size, minimum and maximum inertial weights, and learning factors. In each method, the number of iterations was set to 1000, and the population size was 50 for the test data set. In PSO and CPSO, the minimum and maximum inertial weights were 0.4 and 0.9, respectively. Both weights of learning factors *c*_1_ and *c*_2_ were set to 2. In the tested GA and the proposed HTGA, the probability of crossover (*p_c_*) with an exchange probability was 0.3 and the probability of mutation (*p_m_*) with an exchange probability was 0.05.

### Statistical Analysis

The OR was used to evaluate the risk of an SNP barcode [32], and it was defined as follows:

OR = (*TP* × *TN*) / (*FP* × *FN*) (**6**)

where *TP* represents the number of true positives, *TN* represents the number of true negatives, *FN* represents the number of false negatives, and *FP* represents the number of false positives.

## Results

### Data Sets

A set of 26 SNPs related to growth factor genes was selected to simulate a data set. Several growth factor–related breast cancer genes (*EGF*, *IGF1*, *IGF1R*, *IGF2*, *IGFBP3*, *IL10*, *TGFB1*, and *VEGF*), including 26 SNPs, were used as simulation data to evaluate existing algorithms and the proposed HTGA. The data set only provided the genotype frequencies of each SNP without the original raw data of genotypes. Table 2 presents the SNPs and genotype distributions. The simulated frequencies of SNPs were acquired from the literature [33]. SNPs used in the original data comprised different numbers of individuals; therefore, the number of every SNP must be normalized to the same number. The new data were randomly generated according to the frequency of the original data. All SNP data from the data source were adjusted to 5000 samples for all genotype distributions. For example, for *SNP*1 (gene, *EGF*; dBSNP ref. rs2237054), the total number of three genotypes (ie, TT, TA, and AA) in the control was 2273 (2008 + 259 + 6). The percentage for each genotype in *SNP*1 was calculated as “original data*/sum (%)” (ie, 2008/2273, 88.3% for TT; 259/2273, 11.4% for TA; and 6/2273, 0.3% for AA), where the symbol “*” indicates that the original data were derived from the SNP data set before normalization. On the basis of this percentage, the modified data for *SNP*1 were calculated by multiplying the percentage with the sum of the complete data set (SNP number adjusted to 5000) (ie, 88.3% × 5000 [n=4418] for AA; 11.4% × 5000 [n=569] for Aa, and 0.3% × 5000 [n=13] for aa). Therefore, the modified data for *SNP*1 were adjusted to a total of 5000 (4418 + 569 + 13 = 5000). Thus, 5000 simulation samples of SNP genotypes were randomly generated by following fixed distribution.

**Table 2:** Estimated effect from individual single-nucleotide polymorphisms of 26 growth factor–related genes for the occurrence of breast cancer.

SNP^a^ (gene)	SNP type	Case number/normal number	OR	95% CI	*P* value
1. rs2237054 (*EGF*)	1-TT	4408/4418	N/A^b^	N/A	N/A
1. rs2237054 (*EGF*)	2-TA	570/569	1	0.89-1.14	.97
1. rs2237054 (*EGF*)	3-AA	22/13	1.7	0.85-3.37	.18
2. rs5742678 (*IGF1*)	1-CC	2797/2866	N/A	N/A	N/A
2. rs5742678 (*IGF1*)	2-CG	1844/1837	1.03	0.95-1.12	.52
2. rs5742678 (*IGF1*)	3-GG	359/297	1.24	1.05-1.46	.01
3. rs1549593 (*IGF1*)	1-CC	2924/2970	N/A	N/A	N/A
3. rs1549593 (*IGF1*)	2-CA	1753/1771	1.01	0.93-1.09	.92
3. rs1549593 (*IGF1*)	3-AA	323/259	1.27	1.07-1.50	.008
4. rs6220 (*IGF1*)	1-AA	2643/2698	N/A	N/A	N/A
4. rs6220 (*IGF1*)	2-AG	1933/1951	1.01	0.93-1.10	.80
4. rs6220 (*IGF1*)	3-GG	424/351	1.23	1.06-1.44	.007
5. rs2946834 (*IGF1*)	1-CC	2295/2336	N/A	N/A	N/A
5. rs2946834 (*IGF1*)	2-CT	2171/2150	1.03	0.95-1.12	.53
5. rs2946834 (*IGF1*)	3-TT	534/514	1.06	0.93-1.21	.43
6. rs1568502 (*IGF1R*)	1-AA	2914/2955	N/A	N/A	N/A
6. rs1568502 (*IGF1R*)	2-AG	1840/1807	1.03	0.95-1.12	.46
6. rs1568502 (*IGF1R*)	3-GG	246/238	1.05	0.87-1.26	.65
7. IGF1R-10 (*IGF1R*)	1-AA	3169/3201	N/A	N/A	N/A
7. IGF1R-10 (*IGF1R*)	2-Aa	1545/1582	0.99	0.91-1.08	.77
7. IGF1R-10 (*IGF1R*)	3-aa	286/217	1.33	1.11-1.60	.003
8. rs2229765 (*IGF1R*)	1-GG	1523/1429	N/A	N/A	N/A
8. rs2229765 (*IGF1R*)	2-GA	2533/2489	0.96	0.87-1.05	.33
8. rs2229765 (*IGF1R*)	3-AA	944/1082	0.82	0.73-0.92^c^	.001
9. rs8030950 (*IGF1R*)	1-CC	2737/2745	N/A	N/A	N/A
9. rs8030950 (*IGF1R*)	2-CA	1902/1917	1	0.92-1.08	.92
9. rs8030950 (*IGF1R*)	3-AA	361/338	1.07	0.92-1.25	.41
10. rs680 (*IGF2*)	1-GG	2538/2451	N/A	N/A	N/A
10. rs680 (*IGF2*)	2-GA	2074/2183	0.92	0.85-1.00	.04
10. rs680 (*IGF2*)	3-AA	388/366	1.02	0.88-1.19	.79
11. rs3741211 (*IGF2*)	1-TT	1936/1971	N/A	N/A	N/A
11. rs3741211 (*IGF2*)	2-TC	2367/2269	1.06	0.98-1.16	.17
11. rs3741211 (*IGF2*)	3-CC	697/760	0.93	0.83-1.05	.28
12. IGF2-05 (*IGF2*)	1-AA	2651/2694	N/A	N/A	N/A
12. IGF2-05 (*IGF2*)	2-Aa	1955/1952	1.02	0.94-1.11	.69
12. IGF2-05 (*IGF2*)	3-aa	394/354	1.13	0.97-1.32	.12
13. IGF2-06 (*IGF2*)	1-AA	2160/2162	N/A	N/A	N/A
13. IGF2-06 (*IGF2*)	2-Aa	2237/2284	0.98	0.90-1.07	.66
13. IGF2-06 (*IGF2*)	3-aa	603/554	1.09	0.96-1.24	.21
14. rs2132571 (*IGFBP3*)	1-GG	2415/2407	N/A	N/A	N/A
14. rs2132571 (*IGFBP3*)	2-GA	2163/2157	1	0.92-1.09	.99
14. rs2132571 (*IGFBP3*)	3-AA	422/436	0.97	0.83-1.12	.65
15. rs2471551 (*IGFBP3*)	1-GG	3225/3284	N/A	N/A	N/A
15. rs2471551 (*IGFBP3*)	2-GC	1591/1515	1.07	0.98-1.17	.13
15. rs2471551 (*IGFBP3*)	3-CC	184/201	0.93	0.76-1.15	.54
16. rs2854744 (*IGFBP3*)	1-AA	1538/1469	N/A	N/A	N/A
16. rs2854744 (*IGFBP3*)	2-AC	2487/2475	0.96	0.88-1.05	.39
16. rs2854744 (*IGFBP3*)	3-CC	975/1056	0.88	0.79-0.99	.03
17. rs2132572 (*IGFBP3*)	1-GG	2908/3027	N/A	N/A	N/A
17. rs2132572 (*IGFBP3*)	2-GA	1805/1728	1.09	1.00-1.18	.051
17. rs2132572 (*IGFBP3*)	3-AA	287/245	1.22	1.02-1.46	.03
18. rs3024496 (*IL10*)	1-TT	1218/1235	N/A	N/A	N/A
18. rs3024496 (*IL10*)	2-TC	2533/2549	1.01	0.92-1.11	.90
18. rs3024496 (*IL10*)	3-CC	1249/1216	1.04	0.93-1.17	.49
19. rs1800872 (*IL10*)	1-CC	3059/3017	N/A	N/A	N/A
19. rs1800872 (*IL10*)	2-CA	1660/1722	0.95	0.87-1.03	.25
19. rs1800872 (*IL10*)	3-AA	281/261	1.06	0.89-1.27	.53
20. rs1800890 (*IL10*)	1-TT	1703/1701	N/A	N/A	N/A
20. rs1800890 (*IL10*)	2-TA	2455/2508	0.98	0.90-1.07	.63
20. rs1800890 (*IL10*)	3-AA	842/791	1.06	0.95-1.20	.32
21. rs1554286 (*IL10*)	1-CC	3400/3446	N/A	N/A	N/A
21. rs1554286 (*IL10*)	2-CT	1431/1410	1.03	0.94-1.12	.54
21. rs1554286 (*IL10*)	3-TT	169/144	1.19	0.95-1.49	.15
22. rs1800470 (*TGFB1*)	1-TT	1850/1914	N/A	N/A	N/A
22. rs1800470 (*TGFB1*)	2-TC	2372/2399	1.02	0.94-1.11	.62
22. rs1800470 (*TGFB1*)	3-CC	778/687	1.17	1.04-1.32	.01
23. rs699947 (*VEGF*)	1-CC	1236/1273	N/A	N/A	N/A
23. rs699947 (*VEGF*)	2-CA	2511/2463	1.05	0.95-1.16	.33
23. rs699947 (*VEGF*)	3-AA	1253/1264	1.02	0.91-1.14	.73
24. rs1570360 (*VEGF*)	1-GG	2278/2341	N/A	N/A	N/A
24. rs1570360 (*VEGF*)	2-GA	2214/2132	1.07	0.98-1.16	.13
24. rs1570360 (*VEGF*)	3-AA	508/527	0.99	0.87-1.13	.92
25. rs2010963 (*VEGF*)	1-GG	2354/2279	N/A	N/A	N/A
25. rs2010963 (*VEGF*)	2-GC	2133/2157	0.96	0.88-1.04	.31
25. rs2010963 (*VEGF*)	3-CC	513/564	0.88	0.77-1.01	.07
26. rs3025039 (*VEGF*)	1-CC	3744/3741	N/A	N/A	N/A
26. rs3025039 (*VEGF*)	2-CT	1160/1174	0.99	0.90-1.08	.81
26. rs3025039 (*VEGF*)	3-TT	96/85	1.13	0.84-1.52	.47

^a^SNP: single-nucleotide polymorphism.

^b^N/A: not applicable.

### Comparison of Cases and Controls in Terms of the Effect of a Single SNP

Table 2 compares patients and normal subjects in terms of effect (OR and 95% CI) at a single SNP for growth factor–related genes. Two SNPs within two genes (rs2229765-AA [*IGF1R*] and rs2854744-CC [*IGFBP3*]) showed significant protection associations (rs2229765-AA: OR 0.82, *P*=.001; rs2854744-CC: OR 0.88, *P*=.03) for breast cancer. The minimum and maximum protection associations exhibited ORs of 0.82 and 0.88, respectively, and the other SNPs showed nonsignificant protection associations for breast cancer.

### Comparison Between the Proposed HTGA and Existing Algorithms

We compared PSO [34], CPSO [35], and the GA [24] with the HTGA for 2-SNP to 7-SNP barcodes with protection associations (Table 3). ORs (<1) indicate the impact of the protection association of SNP barcodes for the occurrence of breast cancer. A high difference between cases and controls in the SNP barcodes represents informative protection associations, and *P*<.05 indicates a significant difference for the SNP barcode between cases and controls. The identified 3-SNP to 7-SNP barcodes showed that the HTGA provided values with a greater degree of difference as compared with PSO, CPSO, and the GA, indicating that the HTGA identified relevant SNP barcodes with protection associations more effectively (Table 3). HTGA-identified SNP barcodes showed ORs ranging from 0.755 to 0.870 (*P*=.003) for protection associations with breast cancer. The 2-SNP and 3-SNP barcodes in PSO, CPSO, and the GA showed significant differences between cases and controls (2-SNP: *P*=.003, *P*=.001, and *P*=.03, respectively; 3-SNP: *P*=.04, *P*=.04, and *P*=.002, respectively). The 4-SNP barcodes in CPSO and the GA showed significant differences (*P*=.04 and *P*=004, respectively), and the 5-SNP barcode in the GA also showed a significant difference (*P*=.03). Although CPSO and the GA provided better ORs as compared with the HTGA in all SNP barcodes, the degrees of difference indicated that the SNP barcodes identified by the HTGA were superior to those identified by other methods, and *P* values >.05 indicated that these differences revealed by the models were not significant.

Optimization algorithms have been widely applied to detect relevant high-order SNP barcodes in disease and cancer studies [24,25,34]. Differences between cases and controls are often applied to evaluate the values of SNP barcodes in terms of their fitness function design. As indicated in Table 3, the HTGA effectively identified the relevant protection associations of SNP barcodes for breast cancer. The logistic regression analysis results were strongly validated by the outstanding performance of the HTGA in breast cancer SNP barcode identification. The SNP barcodes detected by the proposed HTGA are simply associations between a barcode and disease, and this type of analysis does not support the inference of causality.

**Table 3:** Estimation of the best protection single-nucleotide polymorphism barcodes for the occurrence of breast cancer as determined by particle swarm optimization, chaotic particle swarm optimization, the genetic algorithm, and the hybrid Taguchi-genetic algorithm.

Order and method		Combined SNP^a^	SNP genotypes	Control number	Case number	Difference	OR	95% CI	*P* value
**2-SNP**									
	PSO^b^	1,8	1-3	941	816	125	0.841	0.76-0.93	.001
	PSO	N/A^c^	Other	4059	4184	N/A	N/A	N/A	N/A
	CPSO^d^	1,8	1-3	941	816	125	0.841	0.76-0.93	.001
	CPSO	N/A	Other	4059	4184	N/A	N/A	N/A	N/A
	GA^e^	1,10	1-2	1926	1823	103	0.916	0.85-0.99	.03
	GA	N/A	Other	3074	3177	N/A	N/A	N/A	N/A
	HTGA^f^	10,17	2-1	1309	1179	130^g^	0.870	0.79-0.95	.003
	HTGA	N/A	Other	3691	3821	N/A	N/A	N/A	N/A
**3-SNP**									
	PSO	8,9,22	3-1-2	269	225	44	0.829	0.69-0.99	.043
	PSO	N/A	Other	4731	4775	N/A	N/A	N/A	N/A
	CPSO	3,8,9	1-3-1	371	319	52	0.850	0.73-0.99	.04
	CPSO	N/A	Other	4629	4681	N/A	N/A	N/A	N/A
	GA	1,8,15	1-3-1	624	527	97	0.826	0.73-0.94	.002
	GA	N/A	Other	4376	4473	N/A	N/A	N/A	N/A
	HTGA	1,10,17	1-2-1	1158	1035	123^g^	0.866	0.79-0.95	.003
	HTGA	N/A	Other	3842	3965	N/A	N/A	N/A	N/A
**4-SNP**									
	PSO	4,8,14,22	2-3-1-2	99	76	23	0.764	0.57-1.03	.08
	PSO	N/A	Other	4901	4924	N/A	N/A	N/A	N/A
	CPSO	10,17,21,23	2-1-1-1	268	223	45	0.824	0.69-0.99	.04
	CPSO	N/A	Other	4732	4777	N/A	N/A	N/A	N/A
	GA	1,10,17,21	1-2-1-1	795	692	103^g^	0.850	0.76-0.95	.004
	GA	N/A	Other	4205	4308	N/A	N/A	N/A	N/A
	HTGA	1,10,17,21	1-2-1-1	795	692	103^g^	0.850	0.76-0.95	.004
	HTGA	N/A	Other	4205	4308	N/A	N/A	N/A	N/A
**5-SNP**									
	PSO	5,6,8,9,26	1-1-3-2-1	91	75	16	0.821	0.60-1.12	.21
	PSO	N/A	Other	4909	4925	N/A	N/A	N/A	N/A
	CPSO	2,4,8,11,18	1-2-3-1-2	44	32	12	0.726	0.46-1.15	.17
	CPSO	N/A	Other	4956	4968	N/A	N/A	N/A	N/A
	GA	1,4,15,17,21	1-1-1-1-1	657	585	72	0.876	0.78-0.99	.03
	GA	N/A	Other	4343	4415	N/A	N/A	N/A	N/A
	HTGA	1,10,17,21,26	1-2-1-1-1	603	520	83^g^	0.846	0.75-0.96	.009
	HTGA	N/A	Other	4397	4480	N/A	N/A	N/A	N/A
**6-SNP**									
	PSO	4,8,15,19,22,24	1-3-2-2-1-3	2	0	2	0.333	0.04-3.20	.34
	PSO	N/A	Other	4998	5000	N/A	N/A	N/A	N/A
	CPSO	3,4,12,16,20,24	1-1-1-2-2-3	28	21	7	0.749	0.43-1.32	.32
	CPSO	N/A	Other	4972	4979	N/A	N/A	N/A	N/A
	GA	1,2,4,6,15,18	1-1-1-1-1-2	276	247	29	0.900	0.75-1.06	.19
	GA	N/A	Other	4724	4753	N/A	N/A	N/A	N/A
	HTGA	1,10,15,17,21,26	1-2-1-1-1-1	394	327	67^g^	0.818	0.70-0.95	.01
	HTGA	N/A	Other	4606	4673	N/A	N/A	N/A	N/A
**7-SNP**									
	PSO	5,8,11,13,14,24,25	1-1-3-1-1-2-1	6	3	3	0.500	0.13-2.00	.33
	PSO	N/A	Other	4994	4997	N/A	N/A	N/A	N/A
	CPSO	10,12,16,17,19,22,26	2-2-2-1-2-2-1	27	20	7	0.740	0.41-1.32	.31
	CPSO	N/A	Other	4973	4980	N/A	N/A	N/A	N/A
	GA	1,2,6,7,10,14,15	1-1-1-3-2-1-1	38	25	13	0.656	0.40-1.09	.10
	GA	N/A	Other	4962	4975	N/A	N/A	N/A	N/A
	HTGA	1,10,13,15,17,21,26	1-2-2-1-1-1-1	185	141	44^g^	0.755	0.60-0.94	.01
	HTGA	N/A	Other	4815	4859	N/A	N/A	N/A	N/A

^a^SNP: single-nucleotide polymorphism.

^b^PSO: particle swarm optimization.

^c^N/A: not applicable.

^d^CPSO: chaotic particle swarm optimization.

^e^GA: genetic algorithm.

^f^HTGA: hybrid Taguchi-genetic algorithm.

^g^The best results in the *n*-SNP barcodes.

## Discussion

### Principal Findings

**Figure 2:**
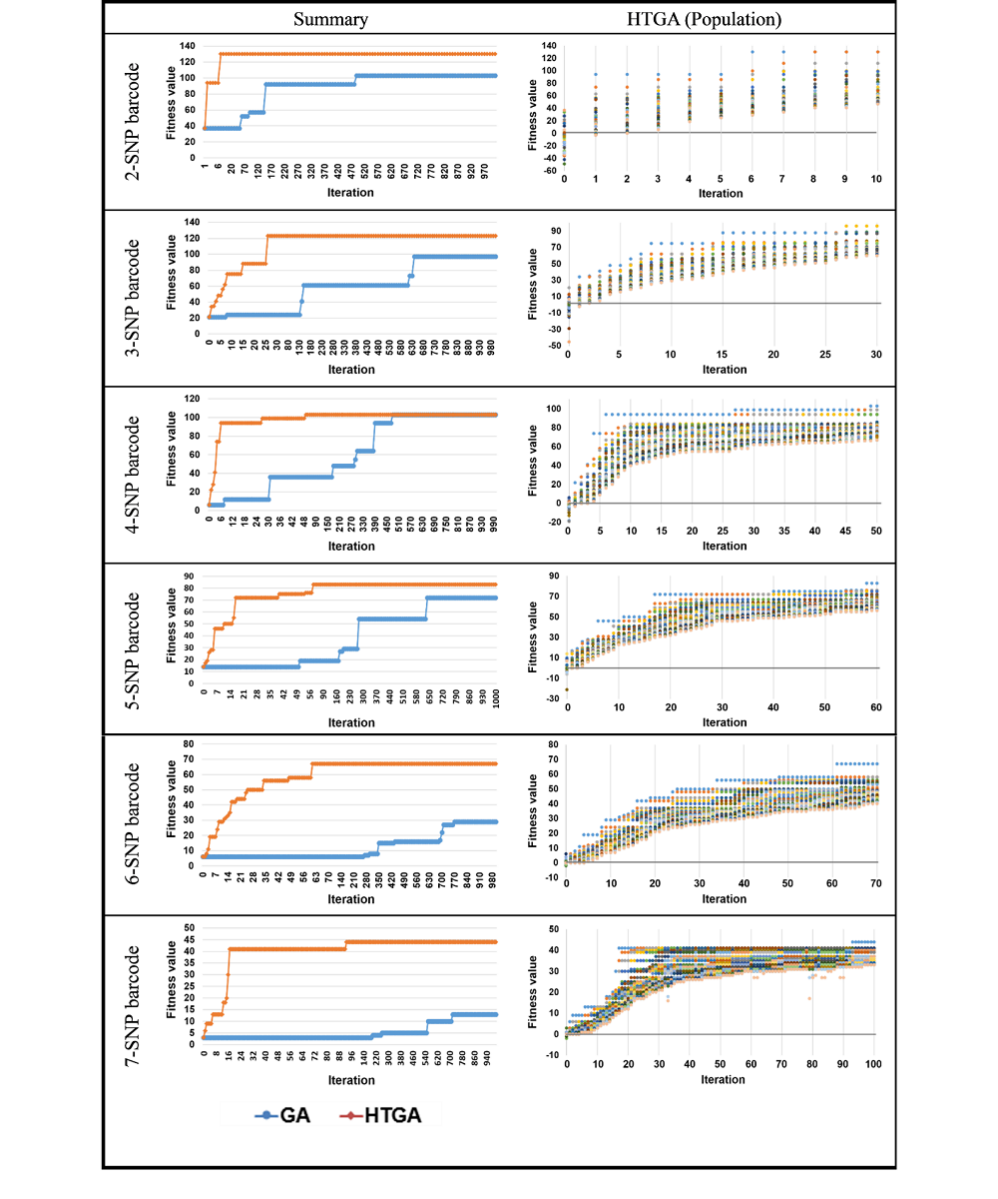
Comparison of improvements to fitness values between the genetic algorithm (GA) and hybrid Taguchi-genetic algorithm (HTGA).The images on the left compare GA and HTGA search results for 1000 iterations. The images on the right present the fitness values of an HTGA population at specific iterations. SNP: single-nucleotide polymorphism.

Many breast cancer studies have identified the associations among the effects of important related genes [36-42], including genes related to DNA repair [43,44] and estrogen-response genes [45]. In this study, we introduced a HTGA to identify the SNP barcodes among 26 breast cancer–related SNPs. The HTGA-generated SNP barcodes were examined to determine their protective effects against breast cancer. The results suggest that nonrelevant SNPs might cumulatively reduce the risk of breast cancer, as indicated by the HTGA-generated preventive SNP barcodes. A search space consisting of SNP barcode combinations can generate numerous local optima in multiple regions. These local optima raise challenges for optimization algorithm search operations, because the heuristic and stochastic properties of such optimization algorithms can easily cause searches to become trapped in local optima. A GA population can be updated by referring to other chromosomes to determine the next position in the search space. However, GA operations can result in stagnation if the chromosomes are similar; points of stagnation in a search space are referred to as local optima. The computational processes and comparisons are shown in Figure 2. A Taguchi system is a nonlinear system with deterministic dynamic behavior owing to its ergodic and stochastic properties. Taguchi methods are used to enhance GA crossover operations, and these methods can be remarkably helpful for avoiding population entrapment in local optima because improved solutions can be found through experimentation. Because the population learns from experience, it can be said to exhibit population intelligence. The HTGA can converge quickly to excellent fitness values for SNP barcodes, whereas the GA is very slow to converge and the results are worse than those of the HTGA (Figure 2), indicating that the GA can very easily result in stagnation in regions that may not include any global optima. However, the population is effectively improved in the HTGA, and Figure 2 shows that the fitness values of chromosomes clearly increase over time, proving that the proposed Taguchi method can be used to improve GA performance to identify SNP barcodes. Moreover, our results prove the ability of this Taguchi-based GA to solve SNP barcode identification problems. The optimal parameters of the HTGA could be further analyzed for enhancing the detection ability of SNP barcodes. Our HTGA includes the probability of crossover and mutation. A further investigation with more data sets is required to determine the optimal parameters. Moreover, selection, crossover, mutation, and replacement operations can be analyzed to determine the superior operation strategy for enhancing the ability of our HTGA to detect potential SNP barcodes. If the HTGA is applied for clinical data, we suggest considering permutation testing to examine the relevance of the results obtained. For each trial in permutation testing, the case/control labels would be scrambled, and the algorithm would then search for an optimal solution. After numerous trials, we would be able to determine the number of times a solution at least as good as the one from the original data is found and then determine if the algorithm is simply fitting the data or identifying underlying associations.

### Conclusions

An HTGA was proposed to effectively identify relevant SNP barcodes among genes related to breast cancer. The study results demonstrated that the HTGA could effectively detect SNP barcodes for problems with numerous high-order SNP barcode combinations. The proposed Taguchi method can improve the GA via the identification of high-dimensional SNP barcodes, and hence, it is integrated following GA crossover operations to systematically optimize chromosomes and thus enhance their values. Moreover, the HTGA can effectively converge to a promising region within the problem space and provide excellent SNP barcode identification. In this study, large data sets were used to evaluate and compare the performances of the GA, PSO, CPSO, and the HTGA, and the results indicated that the HTGA can effectively identify relevant high-order SNP barcodes in breast cancer.

## Abbreviations

CPSO: chaotic particle swarm optimization
GA: genetic algorithm
HTGA: hybrid Taguchi-genetic algorithm
OA: orthogonal array
PSO: particle swarm optimization
SNP: single-nucleotide polymorphism
SNR: signal-to-noise ratio
